# COVID-19 transmission in the first presidential debate in 2020

**DOI:** 10.1063/5.0032847

**Published:** 2020-11-01

**Authors:** Xiaoliang Shao, Xianting Li

**Affiliations:** 1 School of Civil and Resource Engineering, University of Science and Technology Beijing, Beijing 100083, China; 2 Department of Building Science, School of Architecture, Tsinghua University, Beijing 100084, China

## Abstract

The infection risks of Biden, Wallace, and the audience by Trump and the first lady were assessed during the first presidential debate. The debate scene was established numerically, and two cases, i.e., only Trump being infected and both Trump and the first lady being infected, were set up for risk analysis. The infection probabilities at different positions were assessed by using the Wells–Riley equation with consideration of the effects of air distribution and face mask. It was concluded that (1) the infection risks of Biden and Wallace were lower due to the reasonable distance from Trump, with the maximum probability of 0.34% at 40 quanta/h for both Trump and the first lady being infected; (2) the infection probabilities in the audience area were lower for the long distance from the debate stage, with the maximum probability of 0.35%. Wearing masks resulted in a notable decrease in the infection probability to 0.09%; and (3) there was a certain local area surrounding Trump and the first lady with a relatively greater infection probability. The preliminary analysis provides some reference for protection of the next presidential debate and other public events.

## I. INTRODUCTION

The outbreak of Coronavirus Disease 2019 (COVID-19) has attracted global attention, with a total of more than 34 × 10^6^ cases confirmed.^1^ Countries have taken preventive and control measures to deal with the impact of COVID-19.^2^ Unfortunately, however, on October 2, the U.S. president Trump announced he and the first lady had tested positive for coronavirus and were beginning their quarantine and recovery process.^3^ On September 29, Trump held a televised presidential election debate with Biden. According to sources, the debate strictly followed the epidemic prevention standards, and all participants wore masks and kept a social distance.^4^ Although Biden and Trump did not have close contact with each other during the debate, the two presidential candidates and the debate moderator Wallace did not wear masks during the whole process, which inevitably drew people’s attention to the risk of Biden and Wallace being infected with COVID-19.^5,6^

The confirmed transmission routes of COVID-19 are droplet transmission and contact transmission. In recent years, numerous studies have focused on the transport mechanism of the respiratory droplets.^7,8^ In particular during the COVID-19 epidemic, more research focused on this topic. Dbouk and Drikakis^9^ numerically analyzed the transport, dispersion, and evaporation of saliva particles from a human cough. Pendar and Páscoa^10^ investigated the distribution of saliva droplets during sneeze and cough and recommended a safe distance of around 4 m during a sneeze. Das *et al.*^11^ investigated the evolution of droplets under different conditions of temperature, humidity, and wind flow and revealed that the determination of the distance of a healthy individual from an infected person under still air and flowing air relied on different sizes of droplets. Dbouk and Drikakis^12^ and Verma *et al.*^13^ further investigated the effect of masks on airborne droplet transmission and indicated that wearing masks protected the wearer from the droplets from other people, while face shields and a mask with an exhalation valve were insufficient to prevent the droplet transmission. Some researchers are concerned more about the fecal–oral transmission of COVID-19. Li *et al.*^14^ analyzed the impact of toilet flushing on the spread of virus aerosol particles and found that 40%–60% of particles reached above the toilet seat to lead to virus spread. Wang *et al.*^15^ analyzed the particle movement from urinal flushing and found that the particles could reach 0.84 m in 5.5 s, a higher climbing speed than the toilet flushing process.

Recently, studies have shown that COVID-19 may be transmitted through air (or aerosol) especially in a poor ventilated space,^16–18^ which is like other viruses, including influenza, severe acute respiratory syndrome (SARS), tuberculosis, and measles. Efficient air distribution is crucial to the containment of the virus through airborne transmission.^19^ During the debate, Biden and Wallace kept a certain distance from Trump, and there was no direct contact during the whole process, and therefore, the infection risks through direct droplets and contact were reduced. The risk of airborne transmission became the focus of attention. The Wells–Riley equation is a classic model based on the concept of the quantum of infection and reflects the exponential behavior of airborne infections in confined spaces.^20–22^ A quantum indicates the number of infectious droplet nuclei required to infect susceptible persons.^20^ This equation has been used to analyze the outbreaks of measles and TB. In this study, a numerical model of the presidential debate scene was established to simulate the airflow distribution and pollution transmission, and the infection probabilities of people at different positions were assessed by using the Wells–Riley equation. The main objective is to assess the risk of infection from Trump, a confirmed infected person, to the people at different positions in a specific debate hall and ventilation conditions based on the route of airborne transmission, enhancing people’s awareness of the risk of infection in specific indoor spaces.

## II. METHODOLOGY

### A. Calculation method of the infection probability

Based on the assumptions of a well-mixed air space and steady-state conditions, the Wells–Riley equation is as follows:^20,21^

$$P=\frac{C}{S}=1-e^{-Iqpt/Q},$$ (1)

where *P* is the infection probability, *C* is the number of new infections, *S* is the number of susceptible people, *I* is the number of infectors, *q* is the quantum generation rate by an infected person (quanta/h), *p* is the pulmonary ventilation rate (m^3^/h), *t* is the total exposure time (h), and *Q* is the room ventilation rate (m^3^/h).

The actual indoor air is not well mixed. The air temperatures, velocities, and species concentrations among positions are notably different. The non-uniformity factor needs to be included in the probability model. The difference in the quantum concentration between positions essentially reflects the virus dilution ability of the room ventilation to different positions, which is characterized by the so-called “dilution ratio (DR),” defined as the ratio of the quantum concentration in the exhaled breath of infectors to that at the susceptible position,

$$DR=\frac{E_{0}}{E}=\frac{q}{pE},$$ (2)

where *E*_0_ is the quantum concentration in the exhaled breath of infectors (quanta/m^3^) and *E* is the quantum concentration in the inhaled breath of the susceptible person (quanta/m^3^).

The number of quanta inhaled by a susceptible person is

$$Ept=\frac{qt}{DR}.$$ (3)

The infection probability is modified as

$$P=1-e^{-qt/DR}.$$ (4)

Taking the effect of wearing masks into account, Eq. (4) becomes

$$P=1-e^{-qt\left(1-\eta_{I}\right)\left(1-\eta_{s}\right)/DR},$$ (5)

where *η*_*I*_, *η*_*s*_ are mask efficiencies for the infected person and susceptible person, respectively.

The introduction of the DR is more convenient for risk analysis in the non-uniform indoor environment. The DR could be calculated by simulating the transport and distribution of the tracer gas released from the exhaled breath of the infected person. CO_2_ is a commonly used tracer gas and is also produced by the human body and released through the exhaled breath along with the virus. Therefore, it is a good biomarker of the exhaled breath for risk assessment.^22^ In this study, the tracer gas CO_2_ was used to calculate the DR by simulating the distribution of concentration.

### B. Case setup

**FIG. 1.**
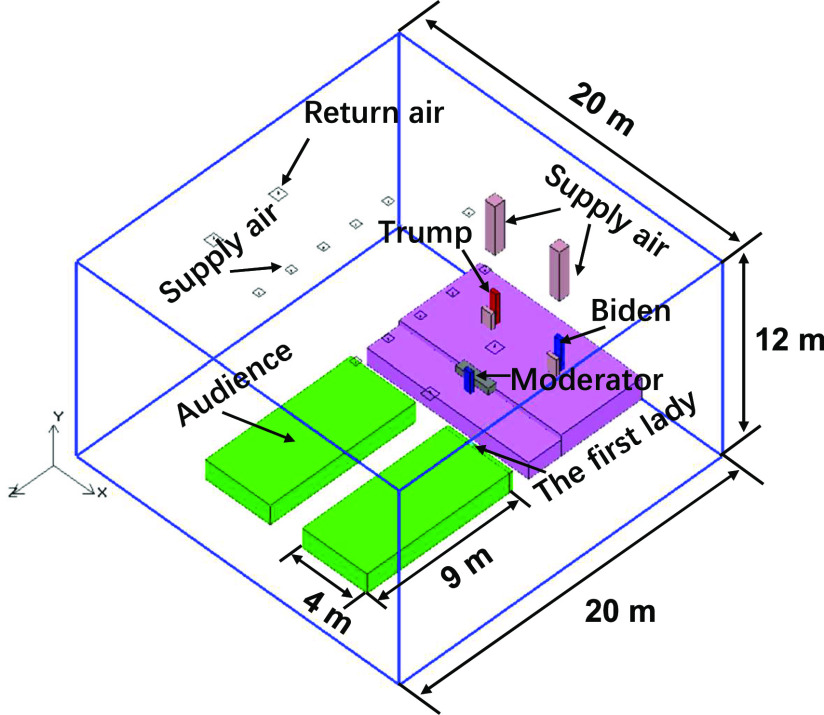
Geometric model of the presidential debate scene.

The geometric model of the debate scene was built up, as shown in Fig. 1.

**FIG. 2.**
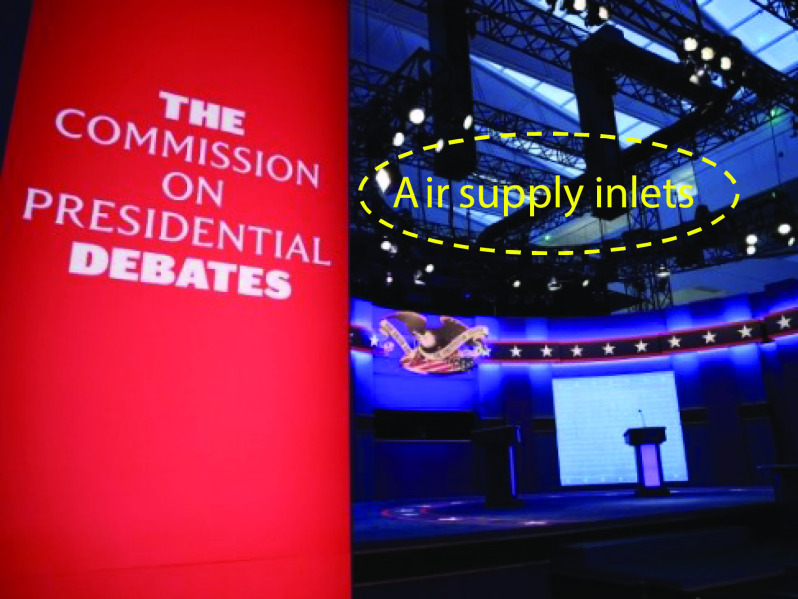
Air supply inlets installed above the debate stage.

Because of the limited information, some assumptions were made on the setting of parameters. The modeling space of the hall was dimensioned as 20 m (length) × 20 m (width) × 12 m (height). The traditional all-air air conditioning system was assumed to be adopted to provide ventilation. The fresh air rate was assumed to be 10 l/s per person according to the conventional design practice. The total air supply rate was assumed to be 6 ACH. No clear air supply inlets and air return outlets were found from the pictures of the site layout, and therefore, the common air distribution of top air supply and top air return was assumed to ventilate the space. A total of 11 air supply inlets (0.4 × 0.4 m^2^) and four air return outlets (0.6 × 0.6 m^2^) were distributed in the ceiling. The air supply velocity was set to 3.73 m/s. According to relevant information, two air supply ducts and inlets (Fig. 2) were temporarily installed above the debate stage, which was supposed to enhance the dilution effect of ventilation on the debate stage. Both air supply inlets were placed 2.65 m above the heads of Trump and Biden. The size of each air inlet was 0.6 × 0.6 m^2^, and the air supply velocity was 2 m/s. Nevertheless, the total air supply rate was in line with the common design scope. The air supply temperature was 22 °C. The total heat release from the audience (Fig. 1) was set to 19.8 kW. The heights of Trump and Biden were set to 1.85 m, while those of the seated Wallace and the first lady were set to 1.3 m and 1.2 m, respectively. The heat release of each of them was set to 75 W. The heat from other potential sources and walls was ignored. According to Wallace, “Trump never approached him and was at least 10 feet away.”^6^ A distance of 3.4 m from Trump in the horizontal direction was set for Wallace. From the live video of the debate, it was estimated that the actual distance between Trump and Biden was greater than 3 m although the distance between their debate tables might be 8 feet. Therefore, a distance of 4 m between Trump and Biden was set. Considering the adverse conditions, the removal effect of the filter on the virus carrying particles in the air handling unit was neglected. During the debate, only Trump, Biden, and Wallace were allowed not to wear masks, while the other people were required to wear masks. Therefore, case 1 was that only Trump released the virus. However, Wallace noticed that at the end of the debate, the first lady did not wear a mask. Since the Trump family and his team did not wear masks during the debate, the first lady might not wear a mask during the whole debate process. Therefore, case 2 was that both Trump and the first lady released the virus simultaneously. The exposure time was 1.5 h, i.e., the debate time. The pulmonary ventilation rate of the infected person was set to 0.3 m^3^/h. The nose was simplified as an opening with a diameter of 0.01 m. The CO_2_ concentration in the exhaled air was 40 000 ppm.^22^ Three quantum generation rates, i.e., 14 quanta/h, 27 quanta/h, and 40 quanta/h, were set to calculate the infection probability.^18^ Because the efficiency of the masks varied between the audience, an average efficiency of 75% was assumed.

### C. Numerical procedure

**FIG. 3.**
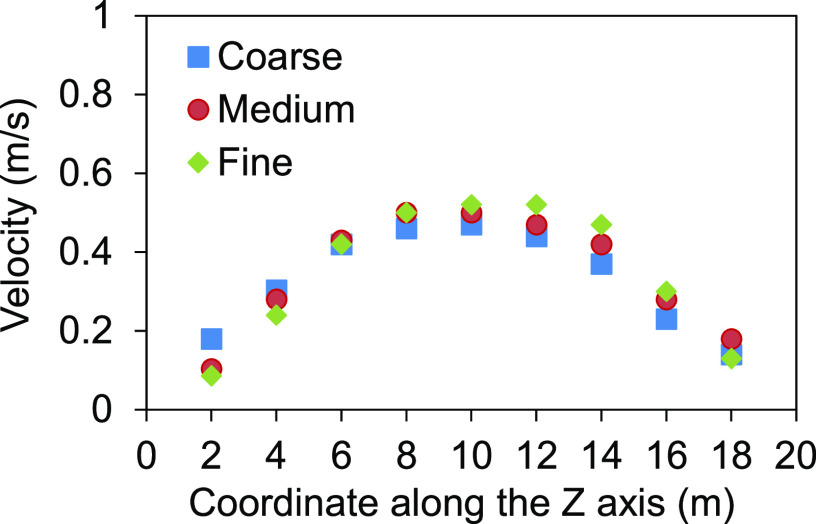
Velocity result for three types of grids.

An indoor zero-equation model specifically for indoor airflow simulations was used to account for the indoor turbulent flow.^23^ The Boussinesq model was adopted to consider the buoyancy effect. The finite volume method was used to discretize the Reynolds-averaged Navier–Stokes equations and mass conservation equations. The body force weighted scheme for pressure and the second-order upwind scheme for momentum, temperature, and CO_2_ concentration were adopted as the discretization scheme. A semi-implicit method for a pressure-linked equation (SIMPLE) algorithm was adopted, and momentum equations were solved on non-uniform staggered grids.^24^ A linear under-relaxation iteration was applied to ensure convergence. The air supply inlets were defined as an opening with a uniform velocity distribution, and the air return outlets were defined as the pressure outlet. In the simulation, CO_2_ was only a tracer gas for predicting the exhaled pollutants of an infector, where the background concentration was set to 0. Because of the existence of the air recirculation in the all-air air conditioning system, the concentration of the tracer gas in the supply air was not 0. Assuming that one air handing unit supplies air to the hall and all the air from multiple air return outlets flows back to the same air handling unit, the concentrations of the tracer gas in the supply air were calculated as 0.83 ppm for case 1 and 1.67 ppm for case 2 based on the mass conservation relationship. The grid independent test was conducted for case 1 and case 2. In case 1, the model room was discretized using 187 173 (coarse), 361 989 (medium), and 586 853 (fine) hexahedral grids, respectively. The velocities in line (X = 10 m, Y = 3 m) were compared for three types of grids, as shown in Fig. 3.

The velocities for 361 989 grids and 586 853 grids were close to each other, while there was a slightly greater discrepancy of 187 173 grids from 586 853 grids at some positions. Therefore, the results from 586 853 (fine) grids were used for analysis. In case 2, there were two infected persons, and 913 392 finer grids were adopted to yield reliable results. For both cases, the areas adjacent to the openings, heat source, and CO_2_ source were refined to accurately reflect the details of air parameters in these locations.

## III. RESULTS AND DISCUSSION

### A. Case 1: Virus was released from Trump

**FIG. 4.**
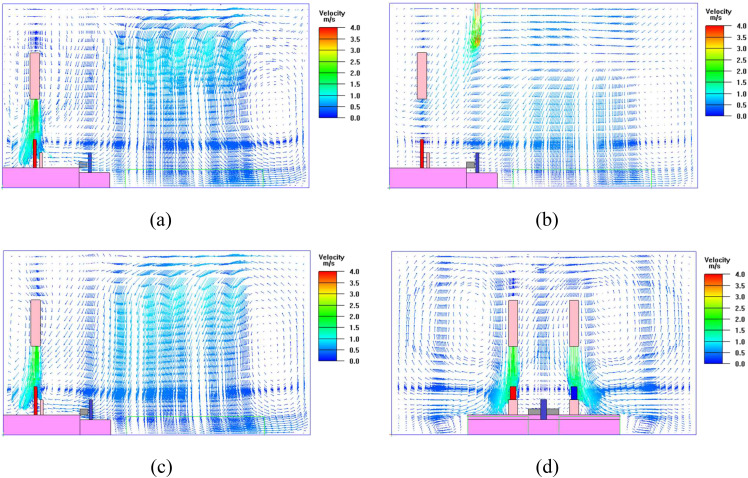
Airflow fields at different planes: (a) across the section of Trump (X = 8 m); (b) across the section of Wallace (X = 10 m); (c) across the section of Biden (Y = 12 m); (d) across the section of Trump and Biden (Z = 2.1 m).

The airflow field in case 1 is shown in Fig. 4.

The airflow could effectively reach the locations of Trump and Biden [Figs. 4(a) and 4(c)]. For Trump, the exhaled virus might be delivered by the airflow to an adjacent area to cause pollution, while for Biden, the upper airflow was expected to provide protection from the virus. The air supply jet path above Wallace bent toward the debate stage [Fig. 4(b)]. There was no clear airflow from Trump to Biden and Wallace [Figs. 4(b) and 4(d)].

**FIG. 5.**
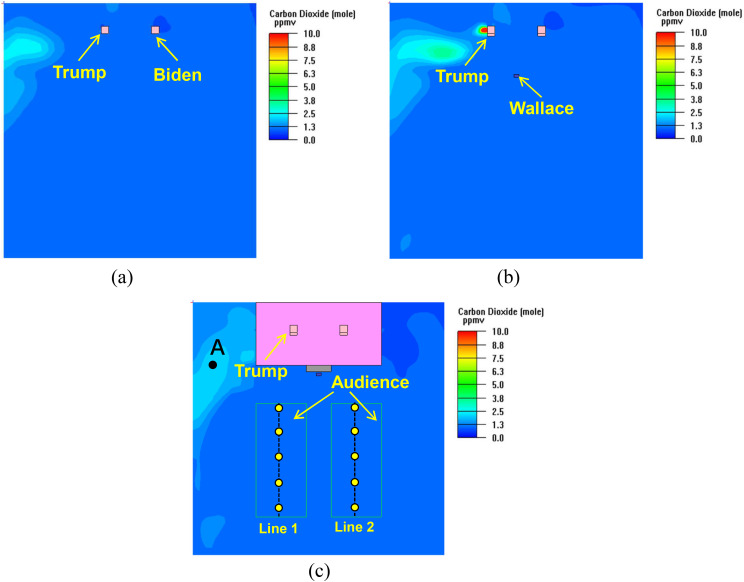
CO_2_ distribution at the respiratory level: (a) Trump and Biden (Y = 3 m); (b) Wallace (Y = 2.15 m); (c) audience (Y = 1.05 m).

The concentration distributions of CO_2_ are shown in Fig. 5. The DRs at the concerned positions are listed in Table I.

**TABLE I.** DRs at various positions in case 1.

Location	DR
Biden	37 736
Wallace	36 036
Location A [Fig. 5(c)]	18 223
Distance from Trump in the left
audience area [line 1 in Fig. 5(c)]
6 m	32 787
8 m	31 923
10 m	31 797
12 m	31 847
14 m	31 949
Distance from Trump in the right
audience area [line 2 in Fig. 5(c)]
6 m	38 132
8 m	37 736
10 m	36 934
12 m	36 364
14 m	35 398

At the respiratory levels of Trump, Biden, Wallace, and the audience, the CO_2_ concentrations were much lower in most of the space. This was because the total ventilation rate for virus dilution was higher in the large space. The relatively greater concentrations occurred in the area around the left side of Trump (Fig. 5). The DRs at the positions of Biden and Wallace as well as in the audience area were more than 30 000, indicating an overwhelming dilution capacity of virus. The DR at location A (1.5 m, 1.05 m, 6 m) [Fig. 5(c)] was 18 223, and the dilution effect of ventilation was reduced.

**FIG. 6.**
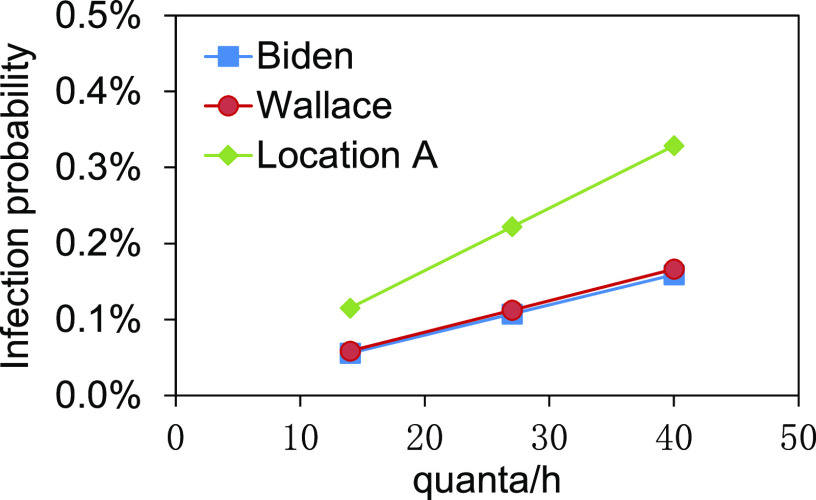
Infection probabilities of Biden, Wallace, and location A.

**FIG. 7.**
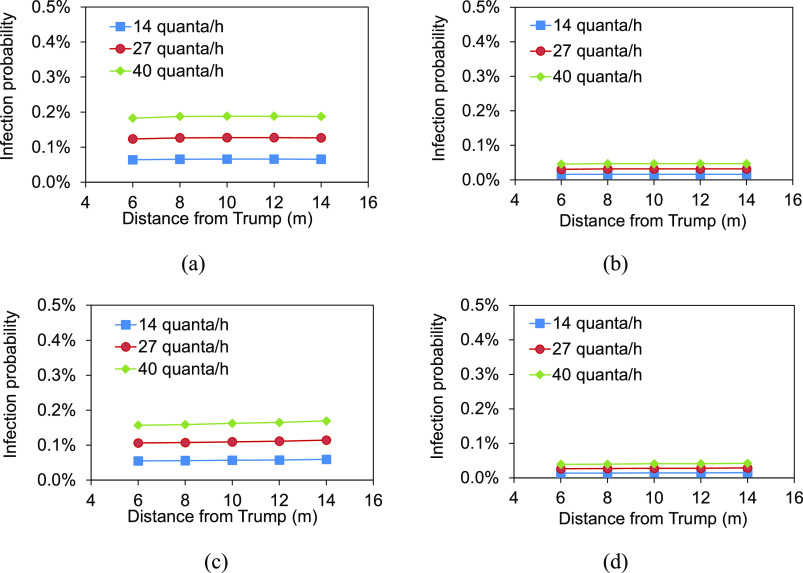
Infection probabilities in the audience area: (a) left audience area, without mask; (b) left audience area, with masks; (c) right audience area, without mask; (d) right audience area, with masks.

Based on the DRs, the infection probabilities at positions of Biden, Wallace, and location A are shown in Fig. 6. The infection probabilities in the audience area are shown in Fig. 7.

The infection probabilities of Biden and Wallace were in the range of 0.06%–0.16% and 0.06%–0.17%, respectively, at different quantum generation rates (Fig. 6). There was a lower risk to be infected. The infection probability at location A close to Trump ranged from 0.12% to 0.33%, higher than those of Biden and Wallace. The audience area was far away from the debate stage, and the infection risk was lower. In the condition that the audience did not wear masks, the infection probabilities in the left audience area were as lower as 0.18%–0.19% at the distance of 6 m–14 m from Trump even at 40 quanta/h [Fig. 7(a)]. The infection probabilities in the right audience area were 0.16%–0.17% [Fig. 7(c)]. There was no significant difference in probability between the audience at different locations. During the debate, all the audience were required to wear masks, and therefore, in this condition, the infection probabilities could be further reduced to 0.05% and 0.04%, respectively, in the left and right audience areas at 40 quanta/h [Figs. 7(b) and 7(d)]. Wearing masks provided powerful protection against virus for the audience. In a word, the infection probabilities for Biden, Wallace, and the audience were lower due to the sufficient ventilation.

### B. Case 2: Virus was released from both Trump and the first lady

**FIG. 8.**
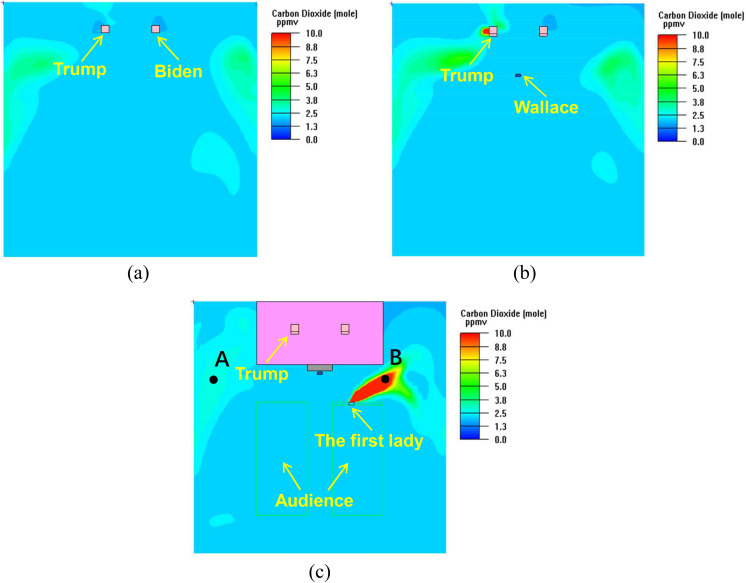
CO_2_ distribution at the respiratory level: (a) Trump and Biden (Y = 3 m); (b) Wallace (Y = 2.15 m); (c) audience (Y = 1.05 m).

When the virus was released from both Trump on the debate stage and the first lady in the first row of the right audience area, the CO_2_ distributions are shown in Fig. 8. The DRs at the concerned positions are listed in Table II.

**TABLE II.** DRs at various positions in case 2.

Location	DR	
Biden	19 324
Wallace	17 699
Location A [Fig. 8(c)]	13 378
Location B [Fig. 8(c)]	3 591
Distance from Trump in the left
audience area [line 1 in Fig. 5(c)]
6 m	17 621
8 m	17 621
10 m	17 467
12 m	17 316
14 m	17 241
Distance from Trump in the right
audience area [line 2 in Fig. 5(c)]
6 m	17 699
8 m	17 621
10 m	17 467
12 m	17 391
14 m	17 316
On the right of the first lady in the first row
1 m	16 260
2 m	17 241
3 m	17 391

In the condition that two infectors existed in the hall, the CO_2_ concentrations increased as a whole (Fig. 8). The DRs at the concerned positions decreased, indicating the dilution effect of ventilation was reduced. The virus release from the first lady had a great influence on the front area. The DR at location B (15.5 m, 1.05 m, 6 m) was 3591, much lower than those at the other positions (Table II).

**FIG. 9.**
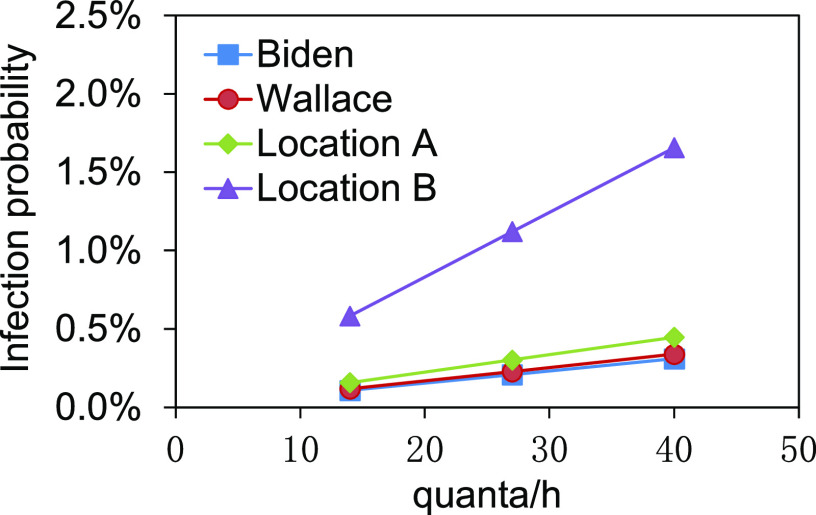
Infection probabilities of Biden, Wallace, location A, and location B.

**FIG. 10.**
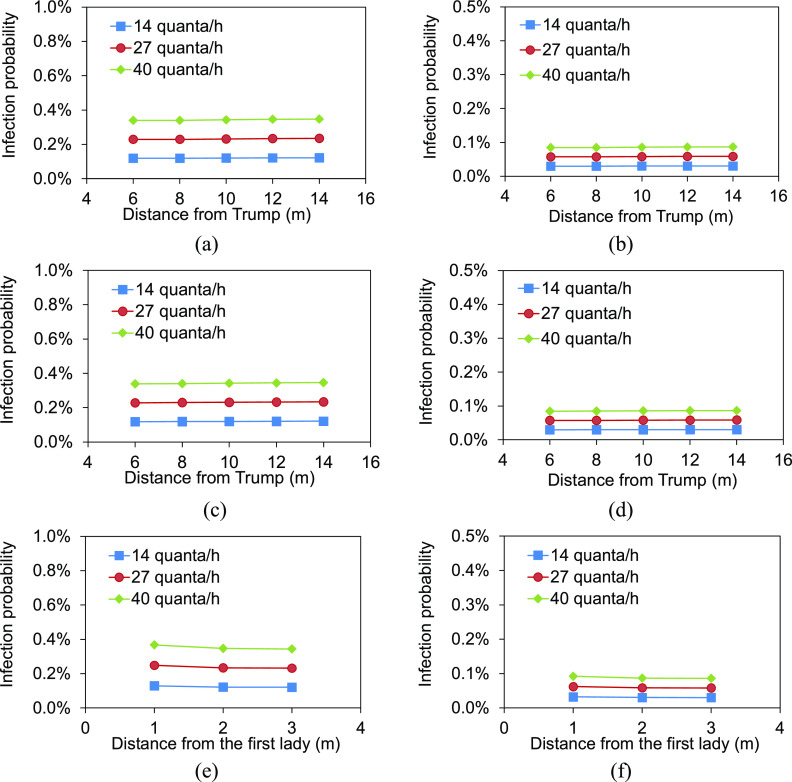
Infection probabilities in the audience area: (a) left audience area, without mask; (b) left audience area, with masks; (c) right audience area, without mask; (d) right audience area, with masks; (e) on the right of the first lady, without mask; (f) on the right of the first lady, with masks.

The infection probabilities at positions of Biden, Wallace, location A, and location B are shown in Fig. 9. The infection probabilities in the audience area are shown in Fig. 10.

Compared with only Trump being infected, both infections of Trump and the first lady resulted in the increased infection probabilities to 0.11%–0.31% for Biden and 0.12%–0.34% for Wallace. However, because both Biden and Wallace kept a certain distance from Trump and the first lady, the infection risks were still lower. The infection probability at location B achieved 1.66% at 40 quanta/h, which was a relatively higher risk with respect to those at the other positions. The audience in the local area surrounding location B would experience a certain infection risk. The existence of two infectors indeed doubled the infection probability in the audience area, with the values of 0.34%–0.35% for those without wearing masks at 40 quanta/h. Therefore, the effect of wearing masks in this case was remarkable, with the probabilities reduced to 0.09%. Even at location B, the infection probability could be reduced to 0.42% when the person in this location wore a mask. The local area surrounding the first lady was the potential high-risk area. During the debate, the Trump family and his team sat together without wearing masks. The infection probabilities of persons seated on the right side of the first lady in the first row were 0.37%, 0.35%, and 0.34%, respectively, at the distances of 1 m, 2 m, and 3 m from the first lady at 40 quanta/h [Fig. 10(e)]. Thanks to the fact that the exhaled virus from the first lady did not accumulate in the surrounding areas, the infection probability in the local area close to the first lady was not significantly higher than that in the other audience area far from the first lady. If the Trump family and his team wore masks, the infection probability could be reduced to 0.09%.

### C. Discussion and recommendations

From analysis of the infection probability of COVID-19, it is found although Biden and Wallace were on the same debate stage as Trump and did not wear masks, their infection probabilities were lower due to the suitable distance from Trump. Increasing the distance between people is recommended especially in the confined space. The infection probabilities in most of the space were much lower for both case 1 and case 2 because of the great fresh air rate in the large space to effectively dilute the virus. Therefore, providing as much fresh air as possible based on the existing ventilation and air conditioning system or opening windows to increase the natural ventilation rate are recommended for virus dilution. For several important positions, it is an effective way to achieve the local protection by installing localized air supply inlets near the positions to deliver fresh air to the person, or adding an air cleaner close to the person to deliver the filtered recirculation air supply. Wearing masks are always the recommended solution to virus protection from both perspectives of preventing the droplet transmission and air (aerosol) transmission. Numerous infected cases happened when people did not wear masks, which reminds us that it is necessary to wear masks especially in the crowed places. Recently, Biden and Wallace have tested negative for COVID-19, which is consistent with our prediction results. Although the possible reason for no droplet transmission and contact transmission cannot be excluded, at least the air (aerosol) transmission is verified to be a potential transmission route to some extent especially in the confined space. Trump and Biden may have a new presidential debate in the following days, and it is hoped that the analysis and recommendations in this study can provide some reference for the follow-up risk prevention and control.

Due to the limited information about the hall structure, related dimensions of facilities, air terminals, etc., some assumptions and simplifications have to be made, which sacrifices a certain accuracy of the results. However, we tried to make the main factors related to the prediction of infection probability such as the relative distance between persons reasonable based on the available information. Therefore, the analysis results are expected to provide guidance for the risk assessment of the similar events.

## IV. CONCLUSIONS

The infection risks of persons at different positions by Trump and the first lady during the first presidential debate were assessed by using the revised Wells–Riley equation. The main conclusions are as follows:

(1) The infection probabilities of Biden and Wallace were lower due to the reasonable distance from Trump, with the maximum probability of 0.17% at the generation rate of 40 quanta/h. In the condition that both Trump and the first lady were infected and did not wear masks, the maximum infection probability increased to 0.34%.
(2) The infection probabilities in the audience area were lower because of the long distance from the debate stage. The maximum infection probabilities were 0.19% and 0.35%, respectively, for only Trump being infected and both Trump and the first lady being infected. Wearing masks resulted in a notable decrease in the infection probabilities to 0.05% and 0.09%.
(3) There was a certain local area surrounding Trump and the first lady with a relatively greater infection probability. Although the overall probability was still low under the sufficient dilution of ventilation in the large space, it is recommended to wear masks to reduce the risk of infection.

For the next presidential debate and other public events, there are some recommendations according to the results of this study: (1) the air conditioning system operates in the all fresh air mode to ensure the maximum dilution of virus; (2) adding more air supply inlets above the debate stage makes the inlets close to the debaters and moderator to enhance the protection; (3) the recommended distance between debaters and the moderator is more than 4 m according to the results; and (4) wearing masks is mandatory for the audience due to the limited distance between each other.

## DATA AVAILABILITY

The data that support the findings of this study are available from the corresponding author upon reasonable request.
